# Polymorphisms in NF-κB Inhibitors and Risk of Epithelial Ovarian Cancer

**DOI:** 10.1186/1471-2407-9-170

**Published:** 2009-06-06

**Authors:** Kristin L White, Robert A Vierkant, Catherine M Phelan, Brooke L Fridley, Stephanie Anderson, Keith L Knutson, Joellen M Schildkraut, Julie M Cunningham, Linda E Kelemen, V Shane Pankratz, David N Rider, Mark Liebow, Lynn C Hartmann, Thomas A Sellers, Ellen L Goode

**Affiliations:** 1 Mayo Clinic College of Medicine, Rochester, MN, USA; 2 H. Lee Moffitt Cancer Research Institute, Tampa, FL, USA; 3 Duke University, Durham, NC, USA; 4 Alberta Cancer Board, Calgary, AB, USA

## Abstract

**Background:**

The nuclear factor-κB (NF-κB) family is a set of transcription factors with key roles in the induction of the inflammatory response and may be the link between inflammation and cancer development. This pathway has been shown to influence ovarian epithelial tissue repair. Inhibitors of κB (IκB) prevent NF-κB activation by sequestering NF-κB proteins in the cytoplasm until IκB proteins are phosphorylated and degraded.

**Methods:**

We used a case-control study to evaluate the association between single nucleotide polymorphisms (SNPs) in *NFKBIA *and *NFKBIB *(the genes encoding IκBα and IκBβ, respectively) and risk of epithelial ovarian cancer. We queried 19 tagSNPs and putative-functional SNPs among 930 epithelial ovarian cancer cases and 1,037 controls from two studies.

**Results:**

The minor allele for one synonymous SNP in *NFKBIA*, rs1957106, was associated with decreased risk (p = 0.03).

**Conclusion:**

Considering the number of single-SNP tests performed and null gene-level results, we conclude that *NFKBIA *and *NFKBIB *are not likely to harbor ovarian cancer risk alleles. Due to its biological significance in ovarian cancer, additional genes encoding NF-κB subunits, activating and inhibiting molecules, and signaling molecules warrant interrogation.

## Background

Despite estimates of more than 21,000 newly diagnosed cases of ovarian cancer and 15,000 related deaths each year in the United States [1], the etiology of ovarian cancer remains poorly understood. Known risk factors include increased risk with family history and use of fertility drugs, and decreased risk with oral contraceptive use, parity, and long duration of breast feeding [2]. Rare, high-penetrant mutations in *BRCA1 *and *BRCA2 *account for approximately 40% of familial risk, leaving most inherited risk unexplained [3,4]. The search for additional loci includes thoughtful selection of candidate genes in key biological pathways, an approach which has been successful in identifying new risk alleles for a variety of cancers [5].

Inflammation has been implicated in ovarian carcinogenesis because of its role in ovulation and post-ovulatory repair. During ovulation the ovarian epithelial surface is damaged, requiring a repair process involving the recruitment of leukocytes and inflammatory cytokines, release of nitrous oxide, DNA repair, and tissue restructuring [6-9]. Over time, this continuous repair of the ovarian epithelial tissue increases the likelihood of errors during replication, potentially leading to carcinogenesis. Nuclear factor-κB (NF-κB) refers to a family of "fast-acting" transcription factors that play a critical role in the inflammatory and innate immune responses [10]. Stimulation by pro-inflammatory cytokines leads to the activation of NF-κB complexes which regulate the expression of key genes controlling apoptosis, angiogenesis, and cell proliferation [10-13]. Aberrant NF-κB functioning can lead to inhibition of apoptosis, constitutive cell replication, and increased angiogenesis, all of which are present in cancer cells [14]. In ovarian cancer, several reports demonstrate the complex relationship between the immune system and established disease, suggesting a role for NF-κB. Immune effectors are thought to assist tumor growth; immunosuppressive regulatory T cells are associated with reduced survival, and the balance of the T cell subsets (regulated by NF-κB) has been shown to be critical to disease outcome [15]. In addition, ovarian tumors acquire aberrant NF-κB functions allowing them to circumvent apoptotic pathways, specifically tumor necrosis factor alpha- (TNFα)-induced apoptosis, and afford protection against environmental insults such as anti-tumor immune effectors or chemotherapy [16-19].

Inhibitors of κB (IκB), IκBα, IκBβ, and IκBε, modulate NF-κB transcription by sequestering complexes of the NF-κB subunits (NF-κB1 [p50/p105], NF-κB2 [p52/p100], RelA [p65], RelB, and c-Rel) in the cytoplasm [10,20]. In response to stimulation by TNFα, interleukin-1 (IL-1), and toll-like receptor (TLR) and T cell receptor (TCR) ligands, IκB proteins are phosphorylated by IκB kinase (IKK) complexes and degraded by the 26S proteasome, allowing for the release and nuclear localization of NF-κB proteins [11,12,21,22]. Improper functioning of IκB proteins can lead to inhibition or constitutive activation of NF-κB [20]. Because of NF-κB's central role in numerous cancer-related processes and involvement in risk of others cancers [23-26], we hypothesized that inherited variation in the genes encoding the key inhibitors IκBα and IκBβ (*NFKBIA *and *NFKBIB*, respectively) is associated with ovarian cancer risk. To examine this hypothesis, we assessed informative single-nucleotide polymorphisms (SNPs) in two case-control study populations.

## Methods

### Study Participants

Participants were recruited at Mayo Clinic in Rochester, MN and at Duke University in Durham, NC. Study protocols were approved by the Mayo Clinic and Duke University Institutional Review Boards, and all study participants provided informed consent. At Mayo Clinic, cases were women over age 20 years with histologically-confirmed epithelial ovarian cancer living in the Upper Midwest and enrolled within one year of diagnosis. Controls without ovarian cancer and without double oophorectomy were recruited from women seen for general medical examinations and frequency-matched to cases on age and region of residence. At Duke University, cases were women between age 20 and 74 years with histologically-confirmed primary epithelial ovarian cancer identified using the North Carolina Central Cancer Registry's rapid case ascertainment system within a 48-county region. Controls without ovarian cancer and who had at least one intact ovary were identified from the same region as the cases using list-assisted random digit dialing and frequency-matched to cases on race and age. Women with borderline and invasive disease were included; cases were 60% serous, 10% mucinous, 14% endometriod, 6% clear cell, and 9% multiple or other histologies. Additional participant details are provided elsewhere [27].

### Data and Biospecimen Collection

Information on known and suspected risk factors were collected through in-person interviews at both sites using similar questionnaires. Mayo Clinic participants had an extra vial of blood drawn during their scheduled medical visit, and DNA was extracted from 10 to 15 mL fresh peripheral blood using the Gentra AutoPure LS Purgene salting out methodology (Gentra, Minneapolis, MN). Duke University participants had venipuncture performed at the conclusion of their interview. DNA samples were transferred to Mayo Clinic and, because of the relatively low quantities of DNA, they were whole-genome amplified (WGA) with the REPLI-G protocol (Qiagen Inc, Valencia CA) which we have shown to yield highly reproducible results with these samples [28]. Genomic and WGA DNA concentrations were adjusted to 50 ηg/μl before genotyping and verified using PicoGreen dsDNA Quantitation kit (Molecular Probes, Inc., Eugene OR). Samples were bar-coded to ensure accurate and reliable sample processing and storage.

### SNP Selection

The selection of informative tagSNPs from among a larger pool of available SNPs allows for maximal genomic coverage and reduced genotyping redundancy [29]. We identified tagSNPs within five kb of *NFKBIA *(chromosome 14q13.2, RefSeq NM_020529.1) and *NFKBIB *(chromosome 19q13.2, RefSeq NM_002503.3) using the algorithm of ldSelect [29] to bin pairwise-correlated SNPs at r^2 ^≥ 0.80 with minor allele frequency (MAF) ≥ 0.05 among publicly-available European-American data from the National Heart, Lung, and Blood Institute's Program for Genomic Applications SeattleSNPs gene-resequencing effort [30]. Within bins of SNPs in linkage disequilibrium (LD), tagSNPs with the maximum predicted likelihood of genotype success (Illumina-provided SNP_Score, San Diego, CA) were selected. Within each gene, we binned 26 SNPs resulting in 13 tagSNPs in *NFKBIA *and eight tagSNPs in *NFKBIB*; four singleton SNPs in *NFKBIA *and two singleton SNPs in *NFKBIB *failed conversion in development of the custom genotype panel and were excluded. The inclusion of additional SNPs with particular suspected functional relevance further increases coverage in a hypothesis-based manner at minimal increased cost; thus, we included all putative-functional SNPs (within 1 kb upstream, 5' UTR, 3' UTR, or non-synonymous) with MAF ≥ 0.05 identified in Ensembl version 34 and Illumina-provided SNP_Score > 0.6, resulting in one additional 3' UTR and three additional 5' upstream SNPs in *NFKBIA*. A total of 13 *NFKBIA *SNPs and six *NFKBIB *SNPs were genotyped (see Additional file 1).

### Genotyping

Genotyping of 1,086 genomic and 1,282 WGA DNA samples (total = 2,368 including duplicates and laboratory controls) on 2,051 unique study participants was performed at Mayo Clinic using the Illumina GoldenGate™ BeadArray assay and BeadStudio software for automated genotype clustering and calling separately for genomic and WGA samples according to a standard protocol [31]. A total of 1,536 SNPs in a variety of pathways were attempted (including *NFKBIA *and *NFKBIB*), and 57 SNPs failed (poor clustering or call rate < 95%). Of 2,051 participants genotyped, 10 were ineligible and excluded, and 74 samples failed (call rate < 90%). Additional quality control (QC) information on the overall panel is provided elsewhere [28]. In *NFKBIA *and *NFKBIB*, 18 of the 19 SNPs were successfully genotyped in both study populations (call rates > 98.9%); *NFKBIA *rs3138050 was excluded for Duke University samples due to poor clustering. For genotype QC metrics see Additional file 1.

### Statistical Analysis

Distributions of demographic and clinical variables were compared across case status using chi-square tests and t-tests as appropriate. Individual SNP associations for ovarian cancer risk were assessed using logistic regression, in which odds ratios (ORs) and 95% confidence intervals (CIs) were estimated. Primary tests for associations assumed an ordinal (log-additive) effect with simple tests for trend, as well as separate comparisons of heterozygous and minor allele homozygous women to major allele homozygous women (referent) using a 2 degree-of-freedom (d.f.) test. In addition, we used a gene-centric principal components analysis to create orthogonal linear combinations of minor allele counts. The component linear combinations that accounted for at least 90% of the variability in the gene were included in a multivariable logistic regression model and simultaneously tested for gene-specific global significance using a likelihood ratio test. Haplotype frequencies were also estimated within each gene and a global haplotype score test of association between haplotypes and ovarian cancer risk was conducted at the gene level using a score test [32]. Individual haplotype tests compared each haplotype to all other haplotypes combined. *NFKBIA *rs3138050 was excluded from gene-level analyses due to failed genotyping in Duke University participants. All analyses were adjusted for age, race, region of residence, body mass index, hormone therapy use, oral contraceptive use, parity, and age at first birth. We used SAS (SAS Institute, Cary, NC, Version 8, 1999), Haplo.stats http://mayoresearch.mayo.edu/mayo/research/biostat/schaid.cfm, and S-Plus (Insightful Corp, Seattle, WA, Version 7.05, 2005) software systems.

## Results

**Figure 1:**
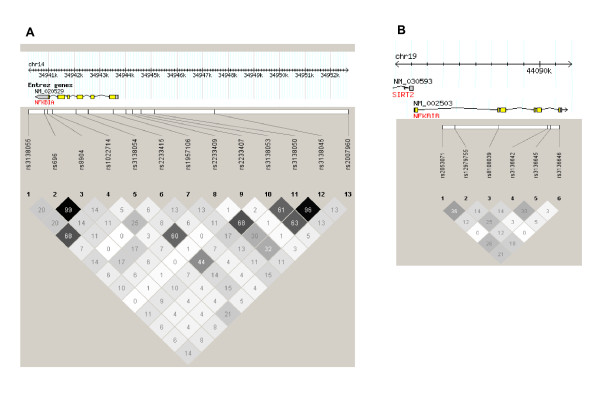
**Linkage Disequilibrium among Study Participants**. (1a). *NFKBIA*; (1b). *NFKBIB*. Haploview 4.1 (Barrett et al., 2005) based on Caucasian controls (N = 941, except N = 462 for *NFKBIA *rs3138050); r^2 ^= 0 = white and r^2 ^= 1 = black; numbers represent r^2 ^* 100, genome build 36.3.

Demographic, reproductive, and lifestyle characteristics of 1,967 epithelial ovarian cancer cases and controls are described in Table 1; generally, the expected distributions in risk factors were observed. As expected given our use of tagSNPs with the inclusion of additional functional SNPs (see Additional file 1), LD (defined as r^2 ^> 0.8) was observed between only a few pairs of *NFKBIA *SNPs and among no pairs of *NFKBIB *SNPs (Figure 1). Risk of ovarian cancer associated with each SNP is provided in Table 2. Only one SNP in *NFKBIA *(synonymous coding SNP rs1957106) showed evidence of association (p = 0.03; adjusted OR, 95% CI: heterozygous 0.77, 0.63–0.94, minor allele homozygous 0.92, 0.65–1.30). Although both ORs are consistent with decreased risk, this over-dominant pattern is unusual and may be due to chance. A second SNP in *NFKBIA *(5' upstream SNP rs3138050) was associated with increased risk assuming a recessive model (minor allele homozygotes v. other genotype groups combined; adjusted OR, 95% CI, 2.24, 1.09–4.61, p = 0.03). This SNP did not adequately genotype in Duke University samples, thus the sample size was limited to Mayo Clinic participants only. No individual SNPs in *NFKBIB *were associated with ovarian cancer risk at p < 0.05. Considering the number of statistical tests, all SNPs lose statistical significance.

**Table 1:** Selected Characteristics of Study Participants

		Mayo Clinic			Duke University		
		
		Cases (N = 396)	Controls (N = 469)	p-value	Cases (N = 534)	Controls (N = 568)	p-value
Age	Mean (S.D.) yrs	59.8 (13.3)	60.1 (13.0)	0.82	54 (11.5)	54.7 (12.2)	0.35
							
Race	White	385 (97.2)	462 (98.5)	0.73	444 (83.3)	479 (84.3)	0.77
	African American	3 (0.8)	2 (0.4)		70 (13.1)	74 (13.0)	
	Asian	2 (0.5)	1 (0.2)		6 (1.1)	2 (0.4)	
	Hispanic	3 (0.8)	3 (0.6)		5 (0.9)	5 (0.9)	
	Native American	0 (0.0)	0 (0.0)		5 (0.9)	6 (1.1)	
	Other	3 (0.8)	1 (0.2)		3 (0.6)	2 (0.4)	
							
Body mass index	< 23 kg/m^2^	79 (20.7)	110 (25.1)	**0.02**	132 (25.4)	139 (25.2)	0.29
	23–26 kg/m^2^	88 (23.1)	121 (27.6)		117 (22.5)	124 (22.5)	
	26–29 kg/m^2^	98 (25.7)	112 (25.6)		106 (20.4)	136 (24.7)	
	≥ 29 kg/m^2^	116 (30.4)	95 (21.7)		165 (31.7)	152 (27.6)	
							
Age at menarche	< 12 yrs	55 (18.7)	68 (15.8)	0.46	130 (24.4)	118 (20.8)	0.44
	12 yrs	77 (26.2)	100 (23.2)		153 (28.8)	166 (29.2)	
	13 yrs	79 (26.9)	126 (29.2)		134 (25.2)	161 (28.3)	
	≥ 14 yrs	83 (28.2)	137 (31.8)		115 (21.6)	123 (21.7)	
							
Oral contraceptive use	Never	176 (47.6)	166 (38.4)	** < 0.001**	182 (34.7)	181 (32.2)	0.36
	1–48 months	98 (26.5)	92 (21.3)		158 (30.2)	160 (28.5)	
	≥ 48 months	96 (25.9)	174 (40.3)		184 (35.1)	221 (39.3)	
							
Postmenopausal	Yes	266 (70.2)	333 (75.3)	0.10	354 (71.7)	372 (67)	0.11
	No	113 (29.8)	109 (24.7)		140 (28.3)	183 (33)	
							
Postmenopausal hormone use	Never	240 (63.8)	248 (58.6)	0.31	196 (37.7)	349 (63)	** < 0.001**
	1–60 months	64 (17)	80 (18.9)		207 (39.8)	109 (19.7)	
	≥ 60 months	72 (19.1)	95 (22.5)		117 (22.5)	96 (17.3)	
							
Parity, n/Age at first birth, yrs	Nulliparous	70 (18.3)	66 (15)	0.07	113 (21.2)	73 (12.9)	**0.003**
	1–2/≤ 20 yrs	29 (7.6)	25 (5.7)		73 (13.7)	69 (12.1)	
	1–2/> 20 yrs	103 (26.9)	131 (29.8)		193 (36.2)	233 (41)	
	≥ 3/≤ 20 yrs	73 (19.1)	64 (14.5)		81 (15.2)	93 (16.4)	
	≥ 3/> 20 yrs	108 (28.2)	154 (35)		73 (13.7)	100 (17.6)	
							
Ovarian cancer family history	Yes	51 (13.3)	33 (7.4)	**0.01**	42 (7.9)	25 (4.4)	**0.02**
	No	333 (86.7)	411 (92.6)		492 (92.1)	543 (95.6)	
							
Ovarian or breast cancer family history	Yes	167 (43.5)	189 (42.6)	0.79	196 (36.7)	190 (33.5)	0.26
	No	217 (56.5)	255 (57.4)		338 (63.3)	378 (66.5)	
							
Smoking, pack years	None	233 (64.9)	285 (68.3)	0.29	297 (57.6)	291 (53.5)	0.41
	<= 20	71 (19.8)	84 (20.1)		130 (25.2)	148 (27.2)	
	> 20	55 (15.3)	48 (11.5)		89 (17.2)	105 (19.3)	
							
Education achieved	No diploma	25 (6.9)	19 (4.3)	** < 0.001**	53 (9.9)	69 (12.1)	0.40
	High school diploma	136 (37.4)	117 (26.4)		153 (28.7)	149 (26.2)	
	Post high school	203 (55.8)	307 (69.3)		327 (61.4)	350 (61.6)	

Data are counts (percentage) unless otherwise indicated. Counts do not total to 1,967 subjects due to missing data for some variables. P-values are from within-sites tests of case-control differences; continuous variables (t-test) and categorical variables (Chi square test). Family history, in first or second degree relative; bold indicates p < 0.05.

**Table 2:** *NFKBIA *and *NFKBIB *Polymorphisms and Adjusted Risk of Epithelial Ovarian Cancer

				General Model OR (95%CI)			Ordinal Model OR (95% CI)	
Gene	SNP ID	bp to next	MAF	AB v AA	BB v AA	p-value	per-allele	p-value
*NFKBIA*	rs3138055	639	0.28	0.99 (0.81–1.20)	1.02 (0.72–1.46)	0.98	1.00 (0.87–1.16)	0.99
	rs696	124	0.38	0.94 (0.77–1.15)	0.85 (0.64–1.14)	0.54	0.93 (0.81–1.06)	0.28
	rs8904	190	0.38	0.93 (0.76–1.13)	0.84 (0.63–1.13)	0.49	0.92 (0.80–1.05)	0.24
	rs1022714	900	0.21	0.94 (0.78–1.15)	0.98 (0.61–1.59)	0.85	0.96 (0.82–1.13)	0.65
	rs3138054	485	0.17	1.18 (0.97–1.44)	0.97 (0.51–1.85)	0.26	1.13 (0.94–1.35)	0.19
	rs2233415	978	0.27	0.84 (0.69–1.02)	0.93 (0.65–1.32)	0.22	0.91 (0.78–1.05)	0.20
	rs1957106	500	0.30	**0.77 (0.63–0.94)**	**0.92 (0.65–1.30)**	**0.03**	0.87 (0.76–1.01)	0.07
	rs2233409	253	0.23	1.11 (0.92–1.35)	0.93 (0.61–1.40)	0.48	1.04 (0.89–1.21)	0.61
	rs2233407	331	0.06	1.27 (0.96–1.70)	0.24 (0.04–1.24)	0.06	1.13 (0.86–1.47)	0.38
	rs3138053	515	0.29	1.03 (0.85–1.25)	1.06 (0.75–1.49)	0.93	1.03 (0.89–1.19)	0.70
	rs3138050	2,352	0.22	1.04 (0.77–1.40)	2.28 (1.10–4.73)	0.09	1.20 (0.94–1.54)	0.14
	rs3138045	5,214	0.22	1.00 (0.82–1.22)	1.25 (0.80–1.97)	0.62	1.05 (0.89–1.23)	0.56
	rs2007960	---	0.39	1.03 (0.84–1.26)	1.02 (0.78–1.34)	0.96	1.01 (0.89–1.16)	0.83
								
*NFKBIB*	rs2053071	886	0.35	0.87 (0.71–1.06)	1.08 (0.80–1.46)	0.22	0.99 (0.86–1.13)	0.86
	rs12979755	3,098	0.39	0.99 (0.81–1.21)	0.99 (0.75–1.31)	0.99	0.99 (0.87–1.14)	0.93
	rs8108039	3,501	0.19	0.99 (0.81–1.21)	1.40 (0.82–2.38)	0.44	1.05 (0.89–1.24)	0.57
	rs3136642	233	0.39	1.03 (0.84–1.26)	1.05 (0.80–1.39)	0.93	1.03 (0.90–1.17)	0.71
	rs3136645	628	0.20	1.02 (0.83–1.24)	1.23 (0.76–1.99)	0.70	1.05 (0.89–1.24)	0.56
	rs3136646	---	0.22	0.99 (0.81–1.21)	0.78 (0.51–1.18)	0.49	0.94 (0.80–1.10)	0.41

Adjusted for race, age, area of residence, body mass index, hormone therapy use, oral contraceptive use, parity, and age at first birth; *NFKBIA *rs3138050 exclude Duke University participants; MAF, minor allele frequency among controls; bold = < 0.05; bp to next represents distance in base pairs between SNPs.

To assess whether overall variation within each gene was associated with ovarian cancer risk, we performed multiple logistic regression for participants with complete genotype data (N = 1,901 for *NFKBIA*, N = 1,930 for *NFKBIB*). Gene-level logistic regression revealed null results (*NFKBIA*, d.f. = 12, p = 0.23; *NFKBIB*, d.f. = 6, p = 0.97) as did the potentially more-powerful logistic regression analysis using principal components (*NFKBIA*, d.f. = 6, p = 0.79; *NFKBIB*, d.f. = 4, p = 0.89).

Haplotype analysis can reveal hidden associations with alleles at ungenotyped variants. Within *NFKBIA*, five haplotypes were estimated to have frequencies > 0.05; no associations were observed with any of these. Three rare haplotypes were associated with increased risk (see Additional file 2); however, overall variation among all haplotypes combined was not associated with risk (p = 0.32). Four *NFKBIB *haplotypes had estimated frequencies > 0.05; no common or rare haplotypes were associated with risk, and overall haplotype associations were null (p = 0.50). In summary, single-SNP, multi-SNP, and haplotype analyses do not indicate that *NFKBIA *or *NFKBIB *harbor risk alleles for ovarian cancer.

## Discussion

To our knowledge, this is the first examination of inherited variation in the NF-κB signaling pathway in relation to epithelial ovarian cancer risk. The two genes studied, *NFKBIA *and *NFKBIB*, encode IκBs with critical roles in regulating NF-κB transcription by directly binding to NF-κB subunits in the cytoplasm. We assessed a comprehensive set of SNPs in these two genes in a large combined case-control study, and found no evidence of association. Strengths of this study include large sample size, choice of candidate genes, use of multiple study populations, LD-based SNP selection, robust genotyping, control of potential confounding variables, and application of a variety of genetic analysis tools. Limitations of this study include the focus on only two genes in a large pathway, the lack of an independent replication outside of the Mayo Clinic and Duke University datasets, and the lack of functional analyses. This study was designed to detect modest genetic associations with ovarian cancer risk; results suggest that common risk alleles of modest effect size may not reside in *NFKBIA *or *NFKBIB*.

Although no association was found here, inherited variation in *NFKBIA *and *NFKBIB *have been associated with increased risk of other cancers including melanoma [26], colorectal cancer [25], multiple myeloma [24], and Hodgkin lymphoma [23]. Considering the vast evidence on the importance of NF-κB in carcinogenesis, additional examination of NF-κB including study of inherited variation in the NF-κB pathway and risk of epithelial ovarian cancer is warranted.

## Conclusion

Study of inherited variation within the NF-κB pathway has the potential to identify risk alleles accounting for the residual increased familial risk of ovarian cancer [3]. The present analysis is an early epidemiologic assessment which indicates that *NFKBIA *and *NFKBIB *are not likely to harbor risk alleles under our statistical assumptions; the key limitation of our study is its focus on only two genes. Other genes to examine are numerous and a more thorough examination of polymorphisms within this pathway is needed to better understand the complexities of ovarian carcinogenesis.

## Abbreviations

NF-κB: nuclear factor-κB; IL-10: interleukin-10; TNFα: tumor necrosis factor alpha; IκB: inhibitors of κB; IL-1: interleukin-1; TLR: toll-like receptor; TCR: T cell receptor; IKK: IκB kinase; SNPs: single-nucleotide polymorphisms; WGA: whole-genome amplification; MAF: minor allele frequency; LD: linkage disequilibrium; QC: quality control; ORs: odd ratios; CIs: confidence intervals; d.f.: degree-of-freedom.

## Competing interests

The authors declare that they have no competing interests.

## Authors' contributions

KLW compiled background material and wrote paper. RAV performed statistical analysis. CMP coordinated gene selection. BLF oversaw statistical analysis. SA performed data management. KLK assisted with results interpretation. JMS provided samples and epidemiologic guidance. JMC performed genotyping. LEK assisted with development of analytical plan. VSP assisted with development of analytical plan. DNR performed SNP selection. ML oversaw control recruitment at Mayo. LCH oversaw case recruitment at Mayo. TAS designed the study, coordinated initial collaboration and obtained funding. ELG developed hypothesis, designed analysis, obtained funding and wrote paper. The final manuscript was read and approved by all authors.

## Pre-publication history

The pre-publication history for this paper can be accessed here:

http://www.biomedcentral.com/1471-2407/9/170/prepub

## Supplementary Material

Additional file 1 **SNP and Genotype Information**. This table provides SNP information, quality metrics, and genotype counts of ovarian cancer cases and controls. Click here for file

Additional file 2 ***NFKBIA *and *NFKBIB *Haplotypes and Ovarian Cancer Risk**. This table provides estimated haplotype frequencies and results of score testing for association with ovarian cancer risk. Click here for file
