# Supplementary material for: Polymorphisms in NF-κB Inhibitors and Risk of Epithelial Ovarian Cancer
Source: BMC Cancer. 2009 Jun 6;9:170. doi: 10.1186/1471-2407-9-170 (PMC2706256; doi:10.1186/1471-2407-9-170)
Supplement: Additional file 1 — SNP and Genotype Information. This table provides SNP information, quality metrics, and genotype counts of ovarian cancer cases and controls. [file 1471-2407-9-170-S1.pdf]

Additional file 1: SNP and Genotype Information

Gene	Rsid	Position	Alleles	LD Bin	Location	HWE p-value	Case Genotype, N			Control Genotype, N		
							AA	AB	BB	AA	AB	BB
NFKBIA	rs3138055	34,940,205	T>C	6	3' downstream	0.42	486	370	73	542	416	78
	rs696	34,940,844	C>T	---	3' UTR	0.86	381	411	130	407	469	157
	rs8904	34,940,968	C>T	4	3' UTR	0.84	385	415	124	407	472	150
	rs1022714	34,941,158	C>T	8	intron	0.15	591	299	36	648	345	40
	rs3138054	34,942,058	G>A	2	intron	**0.04**	602	301	20	708	304	21
	rs2233415	34,942,543	C>T	7	intron	0.55	520	330	78	556	395	83
	rs1957106	34,943,521	G>A	5	synonymous	0.12	510	340	77	500	447	86
	rs2233409	34,944,021	G>A	---	5' upstream	0.41	538	343	48	623	357	57
	rs2233407	34,944,274	A>T	9	5' upstream	0.26	808	119	2	919	108	7
	rs3138053	34,944,605	A>G	1	5' upstream	0.34	444	405	80	509	444	83
	rs3138050	34,945,120	G>A	3	5' upstream	**0.01**	218	154	23	276	179	74
	rs3138045	34,947,472	A>G	---	5' upstream	0.08	547	334	46	626	369	41
	rs2007960	34,952,686	T>A	---	5' upstream	0.14	344	428	154	400	466	165
NFKBIB	rs2053071	44,082,771	C>G	3	intron	0.17	409	399	122	431	479	123
	rs12979755	44,083,657	C>T	1	intron	0.56	343	441	140	383	494	160
	rs8108039	44,086,755	C>T	2	intron	0.17	605	292	33	682	326	29
	rs3136642	44,090,256	A>G	5	intron	0.83	337	437	149	381	485	157
	rs3136645	44,090,489	T>C	4	intron	0.14	582	306	42	660	338	35
	rs3136646	44,091,117	C>T	8	intron	0.07	583	303	44	636	333	63

Position from genome build 36.3; Refseq release 29 (May 4, 2008) *NFKBIA* chromosome 14, *NFKBIB* chromosome 19; HWE p-value calculated using white controls only, bold indicates $p < 0.05$; *NFKBIA* rs3138050 failed for Duke University samples; AA, common homozygotes; AB, heterozygotes; BB, rare homozygotes; LD bin indicates tagSNP status with bin 1 tagging the largest number of SNPs.
