# Supplementary material for: Polymorphisms in NF-κB Inhibitors and Risk of Epithelial Ovarian Cancer
Source: BMC Cancer. 2009 Jun 6;9:170. doi: 10.1186/1471-2407-9-170 (PMC2706256; doi:10.1186/1471-2407-9-170)
Supplement: Additional file 2 — NFKBIA and NFKBIB Haplotypes and Ovarian Cancer Risk. This table provides estimated haplotype frequencies and results of score testing for association with ovarian cancer risk. [file 1471-2407-9-170-S2.pdf]

Additional file 2: *NFKBIA* and *NFKBIB* Haplotypes and Ovarian Cancer Risk

<u>*NFKBIA* (p-value=0.32)</u>

Haplotype	Estimated Frequency	Score	p-value
011000100000	0.166	-1.446	0.148
100101000001	0.105	-0.284	0.777
000010010111	0.090	0.980	0.327
011001000000	0.059	-1.826	0.068
000010010100	0.055	-0.382	0.702
100000000000	0.045	0.444	0.657
000000000110	0.040	-0.508	0.612
000000101000	0.037	-0.220	0.826
100100000001	0.034	-0.850	0.395
011000000000	0.032	1.723	0.085
000000000000	0.029	0.954	0.340
100101000000	0.026	-0.259	0.795
011001100000	0.025	-2.157	**0.031**
011001000001	0.024	-0.343	0.732
011000010111	0.023	-1.091	0.275
000000010111	0.019	-0.232	0.817
100100100001	0.013	-0.402	0.687
000010010101	0.013	1.224	0.221
100000000001	0.013	1.226	0.220
000001000000	0.012	1.644	0.100
011000101000	0.009	2.149	**0.038**
011000000110	0.009	2.168	**0.030**
000010100000	0.008	-0.133	0.894
011000000001	0.008	0.764	0.445
011000100001	0.007	0.255	0.799
100101000110	0.006	-0.420	0.675
011000010110	0.006	-0.071	0.944
011001000111	0.005	-0.611	0.541
000001010111	0.005	0.166	0.868
100100101000	0.005	0.695	0.487
100100000000	0.005	2.463	0.014
100100100000	0.004	0.383	0.702
000000000001	0.004	1.799	0.072
100001000000	0.003	-1.231	0.218
100101010111	0.003	-0.845	0.398
000010000111	0.003	-0.085	0.932
000000010100	0.003	0.128	0.898
100000100000	0.003	0.203	0.839
000010000000	0.003	0.466	0.641
011010010111	0.002	-0.527	0.598
011001101000	0.002	-0.347	0.728
000001000001	0.002	0.122	0.903
000000100001	0.002	0.507	0.612
011001010000	0.002	1.124	0.261
000010010110	0.002	1.827	0.068
011001100000	0.001	-0.125	0.901

<u>*NFKBIB* (p-value=0.50)</u>

Haplotype	Estimated Frequency	Score	p-value
010000	0.348	-0.062	0.950
100001	0.161	-0.366	0.714
100110	0.151	0.472	0.637
001100	0.104	1.518	0.129
001000	0.045	-0.253	0.800
000100	0.044	0.508	0.612
010001	0.029	0.095	0.925
001101	0.028	-1.068	0.285
010100	0.027	-0.810	0.418
100000	0.021	-1.220	0.223
000110	0.017	-1.085	0.278
000001	0.008	-1.387	0.165
010010	0.007	1.164	0.244
010110	0.006	-0.035	0.972
001110	0.005	0.223	0.830
100011	0.005	2.954	0.003
100111	0.003	0.373	0.709
101100	0.002	-0.575	0.565
001111	0.002	-0.144	0.886
100100	0.002	0.108	0.914
100010	0.001	0.596	0.551

NFKBIA **(p-value=0.32)**				***NFKBIB*** **(p-value=0.50)**			
**Haplotype**	**Estimated Frequency**	**Score**	**p-value**	**Haplotype**	**Estimated Frequency**	**Score**	**p-value**
000010010010	0.001	0.907	0.364				
100101000111	0.001	1.339	0.181				

0 represents major allele, 1 represents minor allele; haplotypes based on *NFKBIA* rs3138055, rs696, rs8904, rs1022714, rs3138054, rs2233415, rs1957106, rs2233409, rs2233407, rs3138053, rs3138045, rs2007960, and *NFKBIB* rs2053071, rs12979755, rs8108039, rs3136642, rs3136645, rs3136646; global p-value for overall haplotype association within each gene is provided following gene name.
